# Appropriate empirical antibiotic therapy and mortality: Conflicting data explained by residual confounding

**DOI:** 10.1371/journal.pone.0225478

**Published:** 2019-11-19

**Authors:** Romy Schuttevaer, Jelmer Alsma, Anniek Brink, Willian van Dijk, Jurriaan E. M. de Steenwinkel, Hester F. Lingsma, Damian C. Melles, Stephanie C. E. Schuit

**Affiliations:** 1 Department of Internal Medicine, Section Acute Medicine, Erasmus MC, Erasmus University Medical Center Rotterdam, Rotterdam, The Netherlands; 2 Department of Medical Microbiology and Infectious Diseases, Erasmus MC, Erasmus University Medical Center Rotterdam, Rotterdam, The Netherlands; 3 Department of Public Health, Erasmus MC, Erasmus University Medical Center Rotterdam, Rotterdam, The Netherlands; 4 Department of Medical Microbiology and Immunology, Meander MC, Amersfoort, The Netherlands; Hospital Universitari Bellvitge, SPAIN

## Abstract

**Objective:**

Clinical practice universally assumes that appropriate empirical antibiotic therapy improves survival in patients with bloodstream infection. However, this is not generally supported by previous studies. We examined the association between appropriate therapy and 30-day mortality, while minimizing bias due to confounding by indication.

**Methods:**

We conducted a retrospective cohort study between 2012 and 2017 at a tertiary university hospital in the Netherlands. Adult patients with bloodstream infection attending the emergency department were included. Based on *in vitro* susceptibility, antibiotic therapy was scored as appropriate or inappropriate. Primary outcome was 30-day mortality. To control for confounding, we performed conventional multivariable logistic regression and propensity score methods. Additionally, we performed an analysis in a more homogeneous subgroup (i.e. antibiotic monotherapy).

**Results:**

We included 1.039 patients, 729 (70.2%) received appropriate therapy. Overall 30-day mortality was 10.4%. Appropriately treated patients had more unfavorable characteristics, indicating more severe illness. Despite adjustments, we found no association between appropriate therapy and mortality. For the antibiotic monotherapy subgroup (n = 449), patient characteristics were more homogeneous. Within this subgroup, appropriate therapy was associated with lower mortality (Odds Ratios [95% Confidence Intervals] ranging from: 0.31 [0.14; 0.67] to 0.40 [0.19; 0.85]).

**Conclusions:**

Comparing heterogeneous treatment groups distorts associations despite use of common methods to prevent bias. Consequently, conclusions of such observational studies should be interpreted with care. If possible, future investigators should use our method of attempting to identify and analyze the most homogeneous treatment groups nested within their study objective, because this minimizes residual confounding.

## Introduction

Bacterial infections can result in considerable mortality and have a profound global burden [1–3]. Patients with a severe infection (e.g. sepsis) often present in an acute care setting, such as the emergency department (ED). Initiation of targeted antibiotic therapy in the ED is important in patients with a suspected bacterial infection and is possible when the causative pathogen is proven by cultures with determination of the antibiogram [4]. However, this process usually takes over 24 hours and therefore empirical therapy is initiated in the ED. Appropriate empirical antibiotic therapy (i.e. appropriate therapy) is defined as applying the antibiotic agent which matches *in vitro* susceptibility of the isolated bacteria, but was initially provided without evidence on the causative pathogen or its antibiogram [5]. Clinical practice universally assumes that appropriate therapy improves survival in patients with bloodstream infection (BSI).

Although an overall beneficial outcome of appropriate antibiotic therapy in patients with BSI was demonstrated by meta-analyses [6, 7], studies that did not find lower mortality continued to be published [5, 8–14]. An explanation for these conflicting data is confounding by indication [15], yet this was not investigated in these studies [5, 8–14]. Confounding by indication arises because patients at risk of dying of BSI are more likely to receive broad spectrum antibiotic therapy–thus more often appropriate–as physicians want to ensure appropriateness most in severely ill patients [3]. This results in an imbalance in–measured and unmeasured–patient characteristics (i.e. underlying risk profile) between appropriately and inappropriately treated patients, thereby biasing the genuine relation between appropriate therapy and mortality [16].

The main objective of this study was to examine whether administration of appropriate empirical antibiotic therapy affects 30-day mortality in adult patients with BSI attending the ED, while minimizing bias due to confounding by indication. Subsequently, we focused on methodologically explaining why prior investigators suggested no impact of appropriate therapy on survival.

## Materials and methods

### Study design and setting

We conducted a retrospective cohort study at the Erasmus University Medical Center Rotterdam (Erasmus MC), which is a tertiary university hospital in the Netherlands. We used data from all patients attending the ED with BSI from July 2012 through December 2017. Blood cultures are taken in patients suspected for BSI, and subsequently empiric antibiotic therapy is started. Antibiotic advice is protocolized in guidelines based on local and national prevalence and resistance data [17, 18]. These guidelines provide an advice depending on the suspected source of infection and clinical judgement of severity of disease, e.g. working diagnosis. The Medical Ethics Committee of the Erasmus MC reviewed our study and concluded that it did not fall under the scope of the Medical Research Involving Human Subjects Act and therefore no informed consent needed to be obtained. Our study is thus approved and registered under MEC-2018-1450.

### Selection of participants

Patients were eligible for inclusion if they were at least 18 years of age and had a laboratory proven bacterial BSI at the ED. BSI was defined as presence of a known pathogen in one blood culture or a common commensal (e.g. *Staphylococcus epidermidis*) [19] in at least two blood cultures collected on separate occasions within two days from ED admission [19, 20]. Only the first episode of BSI was included to prevent domination of results by individuals that frequently visited the ED.

### Data collection and processing

We combined electronic databases with data from the ED and the department of Medical Microbiology and Infectious Diseases. The ED database included empiric antibiotic therapy administered during the ED visit, potentially relevant and retrospectively available patient characteristics (serving as proxies for severity of disease), and mortality. Treatment strategy was either no antibiotic therapy, antibiotic monotherapy (if only one drug was administered), or antibiotic combination therapy (if more than one drug was administered). Also, patient charts were reviewed to assess dosage errors. General and demographic patients characteristics collected were: sex, age, arrival (by ambulance or other mode of transportation), triage category (according to the Manchester Triage System) [21], disposition (direct intensive care unit admittance or other), chills [22], vomiting [22], need for vasopressors, suspected site of infection (unknown, respiratory, abdominal, urogenital, skin or soft tissue, intravascular or thorax, central nervous system, other), and origin of infection (nosocomial or community-acquired) [23]. To account for severity of disease we used the first recorded vital signs (i.e. body temperature, heart rate, respiratory rate, systolic blood pressure, oxygen saturation, and consciousness), whether there was need for any supplemental oxygen, and calculated the National early warning score (NEWS) [24, 25] (S1 Methods). Additionally, to account for comorbidity we collected all components of the age-adjusted Charlson comorbidity index (CCI) [26] (S1 Methods). The primary outcome was 30-day mortality, because we expected 30 days to be a biologically plausible window to represent the effect of appropriate therapy on mortality [15]. For mortality data we used municipal death registration records.

The Medical Microbiology and Infectious Diseases database contained data about type of pathogen and their susceptibility (antibiogram) for all positive blood cultures collected at the ED. Blood cultures were performed using the BACTEC system (Becton Dickinson Diagnostic Instrument Systems, Sparks, Md) according to the manufactures protocol. Type of pathogen was identified directly in one milliliter of blood by MALDI-TOF MS analysis (Microflex, Bruker Daltonics, Bremen, Germany). The *in vitro* susceptibility to antibiotic agents testing was performed with VITEK 2 (bioMérieux, Marcy l’Etoile, France). Based on earlier applied antibiotic therapy during ED visit and the established susceptibility of the isolated pathogen, we retrospectively determined the appropriateness of empirical therapy. In accordance with previous studies, no empiric antibiotic therapy, ineffective antibiotic therapy (based on antibiogram or if a dosage error was reported), or not intravenously administered antibiotic therapy (except for antibiotics with high bioavailability, i.e. metronidazole and ciprofloxacin) were all considered inappropriate. The interval of antibiotic administration was adjusted in patients with a glomerular filtration rate less than 30 mL per minute, however, this does not affect the initial dosage of antibiotic therapy administered in the ED [5–13, 15].

### Data analysis and control for confounding bias

For descriptive statistics we examined all patient characteristics among appropriately versus (vs.) inappropriately treated patients. Based on distribution data were tested with an unpaired t-test, chi-squared test, or Fisher's exact test.

We considered patient characteristics as confounders during further analyses if, based on expert knowledge, controlling for the variable would reduce bias when studying the relation between appropriate therapy and 30-day mortality [16]. To improve our propensity score methods, we only included potential confounding variables in our models that were statistically related to outcome, as this decreases variance without increasing bias (S2 Methods) [27].

We conducted inferential statistics to investigate the association between appropriate therapy and 30-day mortality while attempting to control for confounding by indication. Results were presented as odds ratios (OR) with 95% confidence intervals (CI). We handled missing data using multiple imputations. For efficiency purposes we imputed 20 datasets using the chained equations method [28].

To limit confounding by indication, we controlled for measured proxies of disease severity (e.g. arrival mode, triage category, direct intensive care unit admittance, components of NEWS, components of CCI) with multiple statistical techniques. First, we performed a conventional multivariable logistic regression analysis. However, this method is known to fall short in case of confounding by indication [29]. Therefore, secondly, we used propensity score methods. Propensity score methods directly focus on indication for treatment under study and potentially provide more precise estimates in studies in which confounding by indication may occur [29]. We applied three analytical procedures with the obtained propensity scores, namely 1) adjustment by logistic regression, 2) stratification, and 3) inverse probability of treatment weighting (S2 Methods) [30–32]. To assess the impact of potential contaminated BSI (i.e. those with a common commensal on multiple blood cultures), we subsequently performed a sensitivity analysis after exclusion of these patients.

Finally, we attempted to limit confounding bias by selecting patients treated with–appropriate or inappropriate–antibiotic monotherapy. When comparing the total appropriately to inappropriately treated group, we expected various degrees of confounding bias for different treatment strategies (i.e. no antibiotic therapy, antibiotic combination therapy, antibiotic monotherapy). We expected that patients with the lowest disease severity and the lowest risk of dying would more often receive no–thus inappropriate–antibiotic therapy. We also expected that severely ill patients with high chance of dying are more likely to receive antibiotic combination therapy to broaden the spectrum, resulting in more often appropriate therapy. Therefore, when studying the relation between appropriate therapy and mortality in the total population, including these treatment strategies potentially contributes to large heterogeneity between appropriately and inappropriately treated patients, which increases risk of confounding bias. We expected that the subset of patients who received antibiotic monotherapy was the least confounded group with more homogeneous measured and unmeasured confounders.

All hypothesis tests were 2-sided, with a significance level of P < .05. Statistical analyses were performed using R version 3.4.4.

## Results

### Patient characteristics

We identified 1.286 adult patients with a positive laboratory proven blood culture taken at the ED. We excluded 247 patients with recurrent BSI, resulting in 1.039 unique patients of whom 729 (70.2%) received appropriate therapy. In 310 patients therapy was inappropriate: 184 patients received no empiric antibiotic therapy, 115 patients were treated with ineffective antibiotic therapy, and in 11 patients antibiotic therapy was not intravenously administered. Of the patients who were appropriately treated, cefuroxime and gentamicin combination therapy was most often administered. 30-day mortality was 10.4%. We found that 673 (64.8%) patients had a gram-negative BSI. The most frequently isolated pathogens were *Escherichia coli* (32.8%), *Staphylococcus aureus* (10.1%), and *Streptococcus pneumoniae* (8.2%). Patient characteristics are shown in Table 1.

**Table 1:** Patient characteristics in appropriately versus inappropriately treated patients (total population).

Characteristic	Appropriate n = 729 (70.2)	Inappropriate n = 310 (29.8)	P-value
**Sex, male**	425 (58.3)	201 (64.8)	.06
**Age, mean (SD), years**^**A**^	60.9 (15.5)	60.1 (15.9)	.44
**Arrival by ambulance**^**A**^	202 (27.7)	47 (15.2)	< .001
**Triage category, acute/highly urgent**^**A**^^,^^**B**^	205 (29.6)	33 (11.1)	< .001
**Direct intensive care unit admittance**^**A**^	66 (9.1)	8 (2.6)	< .001
**Chills**^**A**^	311 (42.7)	134 (43.2)	.92
**Vomiting**	178 (24.4)	68 (21.9)	.43
**Need for vasopressors**^**A**^	36 (4.9)	5 (1.6)	.02
**Suspected site of infection, unknown**	169 (23.2)	70 (22.6)	.90
**Origin, nosocomial**	384 (52.7)	175 (56.5)	.29
**Antibiotic treatment strategy**			
**Combination therapy**	382 (52.4)	22 (7.1)	< .001
**Monotherapy**	347 (47.6)	102 (32.9)	< .001
**No antibiotic therapy**	0 (0.0)	186 (60.0)	< .001
**Vital signs / NEWS parameters**			
**Body temperature, mean (SD),°C**^**A**^^,^^**C**^	38.4 (1.2)	38.0 (1.1)	< .001
**Heart rate, mean (SD), /min**^**D**^	108 (23.8)	100 (19.6)	< .001
**Respiratory rate, mean (SD), /min**^**A**^^,^^**E**^	24 (8.5)	21 (7.1)	< .001
**Systolic blood pressure, mean (SD), mm Hg**^**A**^^,^^**F**^	125 (28.5)	125 (24.5)	.77
**Oxygen saturation, mean (SD), %**^**G**^	95 (5.8)	96 (2.4)	< .001
**Any supplemental oxygen**^**A**^	339 (46.5)	62 (20.0)	< .001
**Consciousness, not alert**^**A**^^,^^**H**^	96 (15.5)	16 (6.5)	< .001
**NEWS, mean (SD)**	6.0 (3.8)	3.8 (3.1)	< .001
**Comorbidities of Charlson comorbidity index**^**I**^			
**Diabetes mellitus, uncomplicated**	147 (20.2)	53 (17.1)	.29
**Diabetes mellitus, end-organ damage**^**A**^	10 (1.4)	3 (1.0)	.77
**Liver disease, mild**^**A**^	93 (12.8)	47 (15.2)	. 35
**Malignancy, leukemia, lymphoma, localized solid tumor**^**A**^	120 (16.5)	61 (19.7)	.25
**Malignancy, metastatic solid tumor**^**A**^	93 (12.8)	40 (12.9)	>.99
**Chronic kidney disease**^**A**^	124 (17.0)	45 (14.5)	.37
**Congestive heart failure**	96 (13.2)	37 (11.9)	.66
**Myocardial infarction**	103 (14.1)	36 (11.6)	.32
**Chronic obstructive pulmonary disease**^**A**^	95 (13.0)	39 (12.6)	.92
**Perivascular disease**	77 (10.6)	44 (14.2)	.12
**Cerebrovascular accident or transient ischemic attack**^**A**^	115 (15.8)	26 (8.4)	.002
**Dementia**^**A**^	30 (4.1)	6 (1.9)	.12
**Connective tissue disease**	57 (7.8)	20 (6.5)	.52
**Peptic ulcer disease**	17 (2.3)	8 (2.6)	>.99
**Type of isolated pathogen**			
**Gram**-**negative BSI**	457 (62.7)	216 (69.7)	.03

Data are presented as No. (%) unless otherwise indicated. Data in this table is not imputed yet. NEWS, national early warning score; BSI, bloodstream infection.

^A^Confounding variables.

^B^Data on triage category were missing for 50 (4.6%) patients.

^C^Data on body temperature were missing for 9 (0.9%) patients.

^D^Data on heart rate were missing for 24 (2.3%) patients.

^E^Data on respiratory rate were missing for 370 (35.5%) patients.

^F^Data on systolic blood pressure were missing for 20 (1.9%) patients.

^G^Data on oxygen saturation were missing for 43 (4.3%) patients.

^H^Data on consciousness were missing for 175 (16.8%) patients.

^I^Comorbidities with a prevalence below 1% are not presented (i.e. moderate to severe liver disease, acquired immunodeficiency syndrome, and hemiplegia).

Patients receiving appropriate therapy had less favorable measured characteristics than patients receiving inappropriate antibiotic therapy: they more frequently arrived by ambulance (27.7% vs. 15.2%), had higher triage categories (29.6% vs. 11.1%), were more often admitted directly to the intensive care unit (9.1% vs. 2.6%), needed vasopressors more frequently (4.9% vs. 1.6%), and received more antibiotic combination therapy (52.4% vs. 7.1%). In addition, appropriately treated patients had more abnormal vital signs and on average a higher NEWS of 6.0 (± 3.8) vs. 3.8 (± 3.1).

### Appropriate empirical antibiotic therapy and 30-day mortality

**Fig 1:**
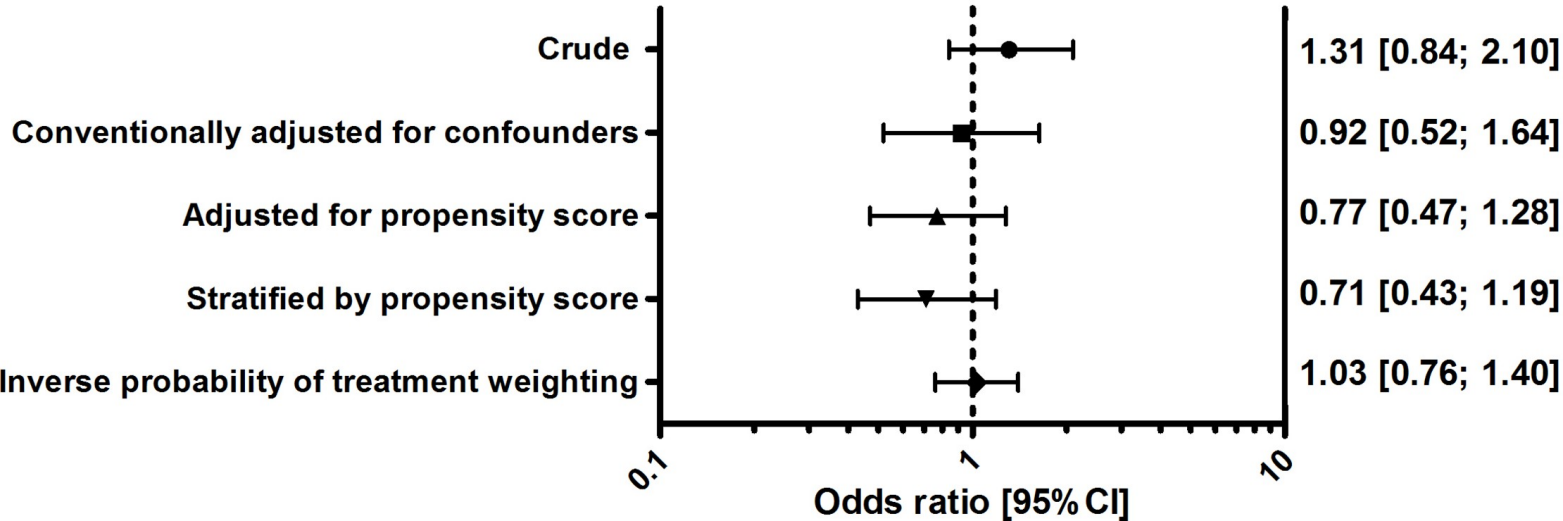
Estimates of appropriate empirical antibiotic therapy on 30-day mortality (total population). CI, confidence interval. Confounding variables: age, arrival, triage category, direct intensive care unit admittance, chills, need for vasopressors, body temperature, respiratory rate, systolic blood pressure, supplemental oxygen, consciousness, diabetes mellitus with end-organ damage, mild liver disease, malignancy, chronic kidney disease, chronic obstructive pulmonary disease, cerebrovascular accident or transient ischemic attack, and dementia. For a detailed description of statistical adjustment techniques, see S2 Methods. This figure shows attenuation of estimates after adjustment for confounders.

Crude 30-day mortality for appropriately treated patients was 11.1% (81 patients) vs. 8.7% (27 patients) for inappropriately treated patients (OR[95%CI]: 1.31 [0.84; 2.10]). There was no association between appropriate therapy and 30-day mortality after conventional adjustment for confounders, adjustment for propensity score, propensity score stratification and inverse probability of treatment weighting (OR[95%CI] ranging from: 0.71 [0.43; 1.19] to 1.03 [0.76; 1.40], Fig 1).

For sensitivity analysis, we examined the impact of excluding patients with common commensal bacteria on multiple blood cultures collected on separate occasions within two days from ED admission. In our study, 24 patients had at least two subsequent blood cultures with a common commensal (17 *Staphylococcus epidermidis*, 3 *Staphylococcus hominis*, 1 *Bacillus licheniformis*, 1 *Rhodococcus equi*, 1 *Staphylococcus capitis*, and 1 *Staphylococcus lugdunensis*). Appropriate therapy was administered in 9 (37.5%) patients. Excluding these patients did not affect our results.

### Subgroup analysis antibiotic monotherapy

There were 449 patients treated with antibiotic monotherapy of whom 347 (77.3%) received appropriate therapy. In 102 patients therapy was inappropriate: 92 patients were treated with ineffective antibiotic therapy and in 10 patients antibiotic therapy was not intravenously administered. Of the patients who were appropriately treated, cefuroxime was most often administered. 30-day mortality was 7.1%. We found that 299 (66.6%) patients had a gram-negative BSI, which is comparable to the rate of gram-negative BSI in the total population (64.8%). The most frequently isolated pathogens were *Escherichia coli* (35.4%), *Staphylococcus aureus* (11.1%), and *Klebsiella pneumoniae* (7.8%). Patient characteristics were comparable for appropriately and inappropriately treated patients, indicating more homogeneity in the monotherapy subgroup compared to the total population (Table 2).

**Table 2:** Patient characteristics in appropriately versus inappropriately treated patients (antibiotic monotherapy).

Characteristic	Appropriate n = 347 (77.3)	Inappropriate n = 102 (22.7)	P-value
**Sex, male**	200 (57.6)	67 (65.7)	.18
**Age, mean (SD), years**^A^	60.1 (15.4)	63.0 (15.1)	.09
**Arrival by ambulance**^A^	55 (15.9)	14 (13.7)	.71
**Triage category, acute/highly urgent**^A^	52 (15.7)	12 (12.2)	.49
**Direct intensive care unit admittance**^A^	10 (2.9)	2 (1.9)	>.99
**Chills**^A^	164 (47.3)	47 (46.1)	.92
**Vomiting**	86 (24.8)	21 (20.6)	.46
**Need for vasopressors**^A^	3 (0.9)	2 (2.0)	.70
**Suspected site of infection, unknown**	86 (24.8)	20 (19.6)	.34
**Origin, nosocomial**	207 (59.7)	63 (61.8)	.79
**Vital signs / NEWS parameters**			
**Body temperature, mean (SD),°C**^A^	38.3 (1.1)	38.1 (1.2)	.05
**Heart rate, mean (SD), beats/min**	103 (20.6)	100 (21.6)	.21
**Respiratory rate, mean (SD), breaths/min**^A^	21 (7.0)	20 (6.4)	.21
**Systolic blood pressure, mean (SD), mm Hg**^A^	128 (25.7)	123 (21.1)	.05
**Oxygen saturation, mean (SD), %**	96 (5.5)	96 (2.3)	.67
**Any supplemental oxygen**^A^	106 (30.5)	33 (32.4)	.82
**Consciousness, not alert**^A^	18 (6.3)	7 (8.5)	.65
**NEWS, mean (SD)**	4.5 (3.0)	4.3 (3.4)	.48
**Comorbidities of Charlson comorbidity index**^**B**^			
**Diabetes mellitus, uncomplicated**	64 (18.4)	17 (16.7)	.79
**Diabetes mellitus, end-organ damage**^A^	5 (1.4)	0 (0.0)	.59
**Liver disease, mild**^A^	53 (15.3)	15 (14.7)	>.99
**Malignancy, leukemia, lymphoma, localized solid tumor**^A^	64 (18.4)	20 (19.6)	.90
**Malignancy, metastatic solid tumor**^A^	45 (13.0)	19 (18.6)	.20
**Chronic kidney disease**^A^	85 (24.5)	21 (20.6)	.49
**Congestive heart failure**	52 (15.0)	11 (10.8)	.36
**Myocardial infarction**	48 (13.8)	16 (15.7)	.76
**Chronic obstructive pulmonary disease**^A^	39 (11.2)	19 (18.6)	.07
**Perivascular disease**	31 (8.9)	13 (12.7)	.34
**Cerebrovascular accident or transient ischemic attack**^A^	57 (16.4)	11 (10.8)	.21
**Dementia**^A^	11 (3.2)	1 (1.0)	.39
**Connective tissue disease**	27 (7.8)	6 (5.9)	.67
**Peptic ulcer disease**	9 (2.6)	3 (2.9)	.88
**Type of isolated pathogen**			
**Gram**-**negative BSI**	214 (61.7)	85 (83.3)	< .001

Data are presented as No. (%) unless otherwise indicated. Data in this table is not imputed yet.

NEWS, national early warning score; BSI, bloodstream infection.

^A^Confounding variables.

^B^ Comorbidities with a prevalence below 1% are not presented (i.e. moderate to severe liver disease, acquired immunodeficiency syndrome, and hemiplegia).

**Fig 2:**
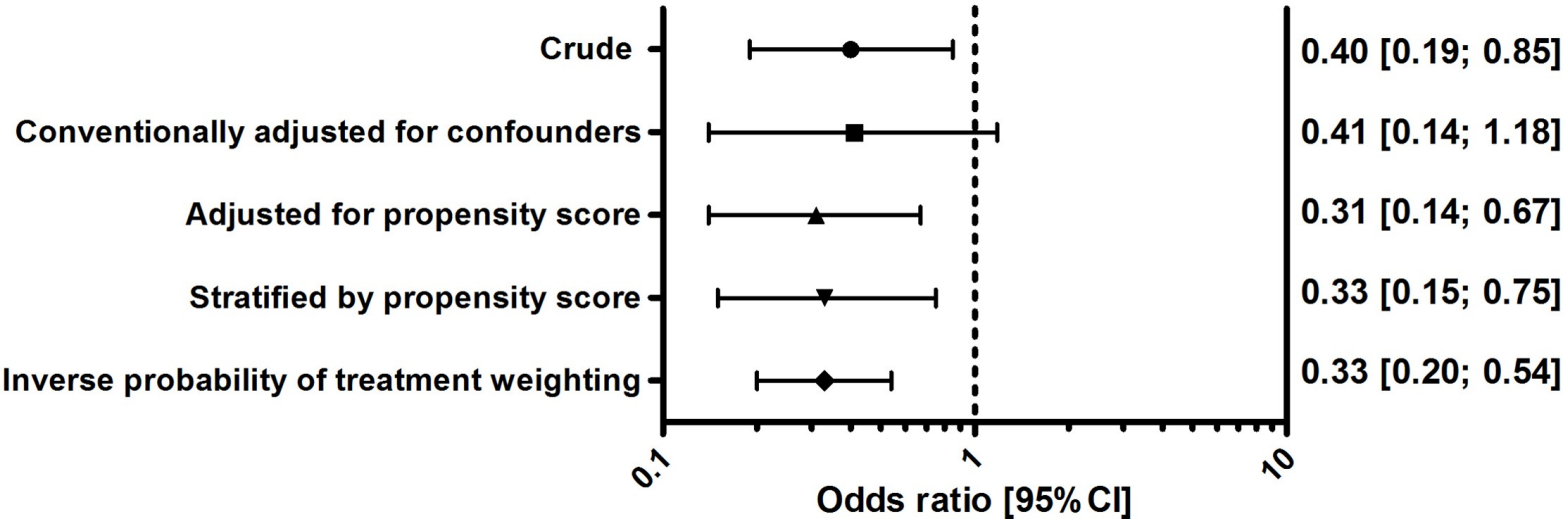
Estimates of appropriate empirical antibiotic therapy on 30-day mortality (antibiotic monotherapy). CI, confidence interval. Confounding variables: age, arrival, triage category, direct intensive care unit admittance, chills, need for vasopressors, body temperature, respiratory rate, systolic blood pressure, supplemental oxygen, consciousness, diabetes mellitus with end-organ damage, mild liver disease, malignancy, chronic kidney disease, chronic obstructive pulmonary disease, cerebrovascular accident or transient ischemic attack, and dementia. For a detailed description of statistical adjustment techniques, see S2 Methods. This figure shows attenuation of estimates after adjustment for confounders.

In the monotherapy subgroup, crude 30-day mortality for appropriately treated patients was 5.5% (19 patients) vs. 12.7% (13 patients) for inappropriately treated patients. Appropriate therapy was associated with lower 30-day mortality after crude estimation, adjustment for propensity score, propensity score stratification, and inverse probability of treatment weighting (OR[95%CI] ranging from: 0.31 [0.14; 0.67] to 0.40 [0.19; 0.85], Fig 2). Conventional adjustment for confounders had an OR with 95%CI of 0.41 [0.14; 1.18].

## Discussion

This study aimed to address the confounding that exists in establishing the effects of antibiotic appropriateness in patients with BSI. Despite extensive adjustment for confounding, we found no association between appropriate empirical antibiotic therapy and mortality when assessing all patients. This finding–in line with previous studies [5, 8–13]–remains counterintuitive and is in contrast to fundamentals of current clinical practice [3].

We hypothesized that confounding by indication was the explanation for finding no association between appropriate therapy and mortality in previous studies. Patients at risk of dying of BSI are more likely to receive broad spectrum antibiotic–thus more often appropriate–therapy as physicians want to ensure appropriateness most in severely ill patients. As a result, the association between appropriate therapy and mortality is biased. In our study, the first clue for confounding by indication was more unfavorable patient characteristics in the appropriately treated group. We noticed this heterogeneity as well in the study of Anderson et al., which also found no association between appropriate therapy and mortality [13]. However, the authors did not consider confounding by indication as a potential explanation for their findings [13]. A second clue for confounding was attenuation of estimates when controlling for bias–with both conventional multivariable logistic regression and propensity score methods. We noticed that in prior studies, that also found no association, there was attenuation of estimates after adjustment for confounders [10, 11]. Since we only adjusted for observed confounders, unmeasured–residual–confounders could still be of potential bias.

Chance of residual confounding is absent in totally homogenous groups (e.g. as in an ideal randomized controlled trial) [16]. Our total population was heterogeneous in measured patient characteristics and we expected various degrees of confounding bias for different treatment strategies. We expected that patients receiving antibiotic combination–thus more often appropriate–therapy were the most ill and patients receiving no antibiotic therapy–thus inappropriate therapy–were the least ill patients. We expected the remainder of patients that received antibiotic monotherapy to be more comparable, as physicians chose to treat these patients presumably based on a more comparable judgment of illness. In addition, the severely confounded treatment strategies–i.e. antibiotic combination therapy and no antibiotic therapy–are per definition excluded during this subgroup analysis. We therefore decided to subsequently analyze the antibiotic monotherapy subgroup. We found that for antibiotic monotherapy measured patient characteristics of appropriately and inappropriately treated patients were more balanced (i.e. homogeneous), lowering the chance of residual confounding. In this subgroup appropriate therapy was associated with lower 30-day mortality. This finding is in line with our expectations and current practice, and supports our hypothesis that residual confounding distorts associations when comparing heterogeneous treatment groups.

Reducing confounding by indication through analyzing homogeneous subgroups–in our study antibiotic monotherapy–is not often done. Previous studies on appropriate therapy and mortality disregarded severely confounded treatment strategies (i.e. antibiotic combination therapy, no antibiotic therapy), which resulted in comparison of heterogeneous groups [5, 8–13]. Therefore, the conclusions of these studies are potentially not trustworthy.

To prevent confounding, we adjusted for validated risk scores (e.g. NEWS, CCI) and applied several adjustment techniques (i.e. conventional multivariable logistic regression and propensity score methods). However, for the total population, these techniques fell short and we were unable to prevent bias. Apparently, a physicians’ decision to initiate a certain therapy is not only based on findings that are represented by such risk score systems, hence statistical adjustment techniques fall short. Thus, conclusions of observational studies comparing heterogeneous groups should be interpreted with care. If possible, future investigators should use our method of attempting to identify and analyze the most homogeneous treatment groups nested within their study objective, as we demonstrated that this minimizes residual confounding.

### Limitations

Our study has limitations. First, we used retrospectively collected data making our study prone to bias. However, the quality of available data was assumed to be high as all data used was essential for daily clinical practice. For only 13 patients (1.3%) documentation was unclear on whether antibiotic therapy was administered at the ED or after discharge, therefore we scored them as inappropriate therapy. We had no data on the exact time to the first antibiotic dose, but only on whether administration was during the ED visit or not. However, timing of antibiotic administration would have had no impact on the outcome of the inappropriately treated group. Delayed treatment might have had an impact on mortality in the appropriately treated group, which could have led to a more extreme estimate than we found already.

Furthermore, we want to emphasize that we considered the association between empiric antibiotic treatment at the ED and 30-day mortality, as this was our main study objective. Depending on disease course and culture results, antibiotic treatment could have been modified later on resulting in a different definitive antibiotic treatment. Also, we had no data on whether any source control such as abscess drainage was performed after ED discharge. Aside from empiric antibiotic treatment at the ED, this may have altered survival as well.

### Conclusions

We initially found that appropriate empirical antibiotic therapy was not beneficial in patients with BSI. We showed that this counterintuitive finding was presumably the result of residual confounding. Patients that present with high disease severity are more likely to receive appropriate therapy than less ill patients. Therefore, the appropriately treated are initially at higher risk of dying than the inappropriately treated. Analyzing these heterogeneous treatment groups results in distorted associations and subsequent conclusions despite the use of common methods to prevent bias. With a subgroup analysis in a more homogeneous population (i.e. antibiotic monotherapy), we found the expected benefit of appropriate therapy. Our study underlines the complexities of performing clinical observational research. In case of heterogeneous groups results should always be interpreted with care. If possible, future investigators should attempt to identify and analyze the most homogeneous treatment groups nested within their study objective, because this minimizes residual confounding.

## Supporting information

S1 Methods Detailed description variables. (DOCX)

S2 Methods Statistical appendix. (DOCX)
