# Supplementary material for: Appropriate empirical antibiotic therapy and mortality: Conflicting data explained by residual confounding
Source: PLoS One. 2019 Nov 19;14(11):e0225478. doi: 10.1371/journal.pone.0225478 (PMC6863559; doi:10.1371/journal.pone.0225478)
Supplement: S1 Methods — (DOCX) [file pone.0225478.s001.docx]

**S1 Methods. Detailed description variables.**

**National early warning score**

We collected all vital signs of the National early warning score (NEWS) [1]: body temperature, heart rate, respiratory rate, systolic blood pressure, oxygen saturation, any supplemental oxygen, and consciousness (AVPU score: alert, voice, pain, unresponsive) [1].

Each vital sign was graded 0-3 [1]. Scores for vital signs were added to obtain a total score. A NEWS over 7 triggers urgent clinical review. See supplementary methods S1 Table 1 for more information about grading of vital signs [1].

**Methods S1 table 1. National early warning score grading.**

| **Grading of vital signs** | **3** | **2** | **1** | **0** | **1** | **2** | **3** |
| --- | --- | --- | --- | --- | --- | --- | --- |
| **Body temperature, °C** | < 35.0 |  | 35.1–36.0 | 36.1–38.0 | 38.1–39.0 | > 39.0 |  |
| **Heart rate, beats/min** | < 41 |  | 41–50 | 51–90 | 91–110 | 111–130 | > 130 |
| **Respiratory rate, breaths/min** | < 91 | 91–100 | 101–110 | 111–219 |  |  | > 219 |
| **Systolic blood pressure** | < 9 |  | 9–11 | 12–20 |  | 21–24 | > 25 |
| **Oxygen saturation** | < 92 | 92–93 | 94–95 | > 96 |  |  |  |
| **Any supplemental oxygen** |  | Yes |  | No |  |  |  |
| **Consciousness, AVPU** |  |  |  | Alert |  |  | Not alert |

AVPU, alert, verbal, pain, unresponsive.

**Charlson comorbidity index**

We collected all comorbidities of the Charlson comorbidity index (CCI) [2]: diabetes mellitus (uncomplicated or end-organ damage), liver disease (mild or moderate to severe), malignancy (leukemia, lymphoma, localized solid tumor, or metastatic solid tumor), acquired immunodeficiency syndrome, chronic kidney disease, congestive heart failure, myocardial infarction, chronic obstructive pulmonary disease, peripheral vascular disease, cerebrovascular accident or transient ischemic attack, dementia, hemiplegia, connective tissue disease, and peptic ulcer disease.

**References**

1. McGinley A, Pearse RM. A national early warning score for acutely ill patients. BMJ. 2012;345:e5310. Epub 2012/08/10. PubMed PMID: 22875955.
2. Quan H, Li B, Couris CM, Fushimi K, Graham P, Hider P, et al. Updating and validating the Charlson comorbidity index and score for risk adjustment in hospital discharge abstracts using data from 6 countries. Am J Epidemiol. 2011;173(6):676-82. Epub 2011/02/19. doi: kwq433 [pii]10.1093/aje/kwq433. PubMed PMID: 21330339.
