# Supplementary material for: Appropriate empirical antibiotic therapy and mortality: Conflicting data explained by residual confounding
Source: PLoS One. 2019 Nov 19;14(11):e0225478. doi: 10.1371/journal.pone.0225478 (PMC6863559; doi:10.1371/journal.pone.0225478)
Supplement: S2 Methods — (DOCX) [file pone.0225478.s002.docx]

**S2 Methods. Statistical appendix.**

**Propensity score methods**

We obtained the propensity score by multivariable logistic regression, with appropriate empirical antibiotic therapy (AEAT) as dependent variable and all confounders as independent variables. The propensity score is a balancing score, ranging from 0 to 1, representing probability of AEAT assignment conditional on observed confounders [1].

We applied three analytical procedures with the obtained propensity score. First, we used the propensity score as single independent covariate representing all confounders during logistic regression. Then we stratified on propensity score by bins of 0.1. For patients within the same bin, distribution of observed confounders is conditionally similar for appropriately and inappropriately treated patients if there is overlap in propensity score. This concept mimics process of randomization. After trimming all patients with non-overlapping propensity scores we obtained odds ratios with standard comparison and performed Mantel-Haenszel pooling [1]. Finally, we used inverse probability of treatment weighting as adjustment technique, which uses the propensity score as a weight during subsequent standard comparison [2].

Based on previous simulation studies, we only included potential confounding variables in our statistical models that were statistically related to outcome (relative risk > 1.3) as this decreases variance without increasing bias [2]. This is mainly important for our propensity score model. Including variables not associated with outcome (30-day mortality), but with exposure (AEAT) can lead to overseparation.

Propensity scores were estimated in our total population and subsequently used in subgroup analyses (i.e. antibiotic monotherapy). Recent simulation studies showed this is a feasible approach [3].

**References**

1. Austin PC. An Introduction to Propensity Score Methods for Reducing the Effects of Confounding in Observational Studies. Multivariate Behav Res. 2011;46(3):399-424. Epub 2011/08/06. doi: 10.1080/00273171.2011.568786. PubMed PMID: 21818162; PubMed Central PMCID: PMC3144483.
2. Brookhart MA, Schneeweiss S, Rothman KJ, Glynn RJ, Avorn J, Sturmer T. Variable selection for propensity score models. Am J Epidemiol. 2006;163(12):1149-56. Epub 2006/04/21. doi: kwj149 [pii]
3. Rassen JA, Glynn RJ, Rothman KJ, Setoguchi S, Schneeweiss S. Applying propensity scores estimated in a full cohort to adjust for confounding in subgroup analyses. Pharmacoepidemiol Drug Saf. 2012;21(7):697-709. Epub 2011/12/14. doi: 10.1002/pds.2256. PubMed PMID: 22162077; PubMed Central PMCID: PMC3383902.
